# Quarterly Summary

**Published:** 1856-04

**Authors:** 


### QUARTERLY SUMMARY.

### ANATOMY AND PHYSIOLOGY.

1.—*Functions of the Spleen.*—Recent researches, as our readers are well aware, have led to a more settled notion of the functions of this mysterious organ, though many important questions relating to them are still in dispute. The names of Gray and Kölliker are conspicuous among those who have contributed to our knowledge of its structure and functions.

The opinions generally entertained may be briefly summed up as follows: The spleen is considered as a storehouse of nutritive material, and as a workshop for fitting the albuminoid matter it receives, during digestion, for the use of the system. It is also thought to have some agency in manufacturing blood corpuscles, as well as in promoting their disintegration. It has long been regarded as a *diverticulum* for blood accumulating in the system, especially in the portal vein. The enlargement of the organ in intermittent fever, which induces congestion of all the abdominal viscera, its periodic swelling during the act of digestion, accounted for by the sudden addition of a large quantity of material to the blood, and its increase during all obstructions to the portal circulation, whether natural or artificial, lead to this conclusion. In addition to this, some cases recently recorded in the Medical Times and Gazette, of London, are exceedingly interesting, in relation to this view of the functions of the spleen, as it looks to the general vascular system, as well as the immediately adjoining veins. They are cases of enlarged spleen during suppressed menstruation, the chief dilatation occurring at the time the monthly flow should have manifested itself, but continuing, like most morbid action, after the first exciting cause had ceased to act.

M. Vulpian, however, (as quoted by the *Virginia Medical Journal* from the reports of the *Societe de Biologie*.) has lately submitted a memoir on the effects of removing the spleen from a dog, which go to show that the loss of the spleen is not very severely felt. The animal lived six years and a half after the operation, without any appreciable derangement of health. Shortly before his death, his blood was examined with reference to its relative proportion of white and red corpuscles. This was found to be normal, when compared with that existing in the blood of a dog upon which no operation whatever had been performed.

The necropsy revealed no increase of the thyroid bodies or the supearenal capsules, which ought to have been hypertrophied, if they were engaged in the same blood-renovation as the spleen, as is usually supposed. The liver was healthy, and full of sugar. There was disease of the heart and ossification of the aorta, which, however, can hardly be supposed to be at all connected with the operation. The spleen was gone, not a trace of it was to be found.

To give greater completeness to the account of the present state of opinions on the functions of this mysterious organ we make an abstract of Mr. H. Gray's views, which we abbreviate from Dr. Carpenter's review of his work, in the British and Foreign Medico-Chirurgical Review for January of last year.

The *development* of the spleen takes place from a distinct centre, and is not as some anatomists suppose, evolved from the same mass with the pancreas. In the fœtus, the history of the development of this organ goes to show that its function is not mainly exercised during intra-uterine life; that it is not *then* either a blood forming or a blood destroying gland. A process of cell-growth goes on while the arteries are being formed, and one of cell-destruction during the development of the splenic vein. From this it is inferred that some secretion is collected in the gland ready to be removed by the veins as soon as they are developed. In the chick, the Malpighian vesicles are not developed till near the end of incubation. The development of the splenic vein does not take place till after green bile has been secreted by the liver, so that Kölliker's notion, that the coloring matter of the bile is formed entirely at the expense of blood-corpuscles which have been disintegrated in the spleen, cannot be adopted.

The weight of the spleen differs at different ages. Towards the end of fœtal life it is 1-300 of the weight of the body, while during adult life it varies from 1-320 to 1-400. In old age it is only 1-700. Hence it follows that the spleen exerts its peculiar function most energetically during the most active period of growth and nutrition.

As to its relations to digestion, he found that its weight increases considerably near the close of this process, or when the new material is becoming or has become blood, and that it decreases considerably after the final completion of that process.

In his description of the *anatomical structure* of this organ, he generally coincides with previous observers. The muscular fibres of the trabeculæ, according to him and many others, possess very feeble contractile properties. In most animals no contractions were produced, while in dogs and cats there was only a slight wrinkling of the surface.

The capillaries terminate sometimes directly in the veins which are dilated at the point of union, but oftener open into intercellular spaces with which the veins are connected. The veins originate (1) by direct continuity with the arteries, (2) by lacunar spaces, (3) by ideal pouches, the second being by far most frequent. They expand very rapidly, and are extremely distensible.

Mr. Gray repeats what had already been said by others that there is a continual process of cell development, growth and decay, going in the pulp of the spleen, accompanied by the formation of nuclei, around which blastema and cells are formed, and which ultimately disappear with these enclosing structures. He adds, however, from his own observations, that these parenchyma-cells are in direct proportion to the activity of nutrition and are particularly frequent when the addition of new material exceeds the waste of the body, whereas, when waste predominates over repair, they are not to be found at all. These parenchyma-cells closely resemble the colorless corpuscles in the blood of the splenic vein. The colored components of the spleen are affirmed by Mr. Gray to be red corpuscles variously modified.

The blood discs found among the parenchyma-cells of the spleen are regarded by this observer as proper constituents of the pulp. These blood discs are usually in various stages of disintegration, till they are found at last reduced to mere granules. Sometimes, however, they exist in their normal condition, invested by a non-nucleated cell. The blood-corpuscle-holding cells, are large, spherical or oval, containing a variable number of altered blood discs and pigment-granules derived from their destruction. These granules are also dispersed through the pulp, together with reddish crystalline forms.

The quantity of these bodies present was influenced by the state of nutrition of the animals, being most abundant when that was highest.

*Chemically*, the chief component of the spleen pulp has an albuminous character. The coloring matter is identical with hæmatin. The reddish crystalline bodies seem to consist of Vischow's hæmatoidin. No relation was traced between the coloring matter of the spleen and that of the bile. Of iron there was 16 per cent. in the ash, and of phosphoric acid 40 per cent., a decided indication of the derivation of the spleen-substance from blood corpuscle. Lactic acid was found, but neither uric acid nor hypoxanthrei.

The *Malpighian corpuscles* are invested with a distinct fibrous membrane derived from the sheaths of the arteries and nourished by fine capillary vessels which ramify over their surface and penetrate their interior. Their contents are granular blastema, nuclei and nucleated cells, corresponding perfectly with the colorless portions of the spleen. Hence Mr. Gray supposes that the same processes of cell-development, cell-growth and cell-destruction go on in the corpuscles as in the pulp.

He found the size of these corpuscles to correspond very exactly with the state of nutrition in the animal experimented on, the maximum of size being attained in highly fed animals, while in the ill-fed, the corpuscles were smaller, and, in some instances, almost entirely absent. Hence it may be supposed that these corpuscles store up the surplus albuminous aliment. Furthermore, during the latter part of the act of digestion and a short time after its completion, they are most abundant, while long after or just before the commencement of this process they are fewest. The nature of the diet also affected them. Thus, when cats were fed on boiled white of egg, the whole spleen acquired a large size, and the Malpighian corpuscles were peculiarly obvious, while, when fed on fat, lean meat, or gelatin, the spleen dwindled to less than one-half its size and the corpuscles became so small as to be invisible to the naked eye. The supply of water modified the size of the corpuscles and the fluidity of their contents.

Remell, Leydig and Huxley state, that the wall of the corpuscles is not separated by any distinct line of demarcation from its contents or the surrounding pulp. They, therefore, regard them as portions of spleen pulp more or less isolated from the rest.

The absolute quantity of *blood* varies with the day. It is greatest about 16 hours after eating; least, about 48 hours after food. In inanition, it rapidly diminishes, and all obstructions of the liver, heart or lungs, greatly augment the size of the organ. In *diving* animals the spleen is very large, an interesting fact in view of the theory of its acting as a diverticulum.

The splenic blood was shown by the *microscope* to contain particles of blood, altered like those found in the organ; *occasionally* corpuscle-holding cells; *almost always*, pigment-granules and always large quantities of white corpuscles. The solid matter in blood of the splenic is *less* than that of common venous blood, the red corpuscles not more than half as numerous, the fibrin and albumen both increased, especially the former. The serum of splenic blood has a reddish-brown tinge.

He believes the function of this organ to be; 1, the modification of the constituents of the blood-corpuscles; 2, the formation of a sort of "sinking fund for albuminous materials." The spleen, therefore, according to him, balances both the quantity and the quality of the blood. He acknowledges the slight effect produced by the extirpation of this organ and says that in some of the animals on which he operated, the process of nutrition seemed to go on more vigorously after the extirpation than before. Dr. Carpenter thinks the author proves too much, and is inclined to believe the change, in the blood, which he describes, accidental, arising from a stagnation or retardation of the circulation in this organ.

---

2.—*Pancreatic Juice.*—Schmidt has recently been experimenting on fresh pancreatic juice, allowed to flow through a fistula. Some interesting facts were ascertained relative to the influence of the operation upon the secretions. The fluid collected immediately after the establishment of the fistulous opening contained six times as much solid matter as that which escaped afterwards. The fluid obtained from the permanent fistula is transparent, colorless, alkaline in its reaction and having a specific gravity of 1.010 to 1.011. At the temperature of 98.6° it instantaneously converts starch into gum and sugar. Schmidt repeats the statement that two per cent. of the secretion would be sufficient to convert all the starch consumed in twenty-four hours into sugar. He, therefore, believes that the chief function of this fluid is to assist the intermediate circulation of fluids, and to saturate the acid of the chyme.

3.—*Saliva.*—Kölliker and Müller confirm the statement, long since made, that the secretion of any single salivary gland, alone, is not sufficient to convert starch into sugar. The mixture of all the secretions, in the human saliva, is admitted, however, to act with great rapidity in producing this change; and the discharge from the mouth of a patient, laboring under mercurial stomatitis, is said to saccharify even more rapidly than normal saliva. When saliva is mixed with gastric juice, in equal proportions, the conversion of starch into sugar does not take place, but when there is an excess of saliva, saccharification goes on.

This cannot be supposed to confirm the opinion of Bernard that the gastric stops the action of the saliva, for a great portion of the starch is undoubtedly converted into sugar, before the aliment reaches the stomach, and when it arrives there, the action of the gastric juice will be mainly confined to the surface, while the saccharification will go on rapidly in the centre.

4.—*Tape Worm.*—It will be remembered that some years since, Siebold and Van Beneden dispelled the obscurity resting over the generation of intestinal worms by showing good reasons for the belief that these parasites were taken in with the food. They found that the different forms, in certain cases, were only different stages of development of the same animal; that the cysticercus of one creature became afterwards the tænia of another which preyed upon it. Some doubt however, still rested over the subject, and many could not understand how the discovery of this change in the lower animals could explain the development of tape worm. Küchenmeister has just completed the demonstration by showing the actual change of cysticered into tænia in the human subject. Cysticerci were administered to a criminal under sentence of death, from 72 to 12 hours before his execution. After death, no less than ten young tape worms were found in the man's duodenum.

5.—*Influence of the Spinal Cord on the Movements of the Iris.*—An interesting case was presented to the Medico-Chirurgical Society, of Edinburgh, at a recent meeting. A patient had aneurism of the subclavian artery pressing backwards, and consequently compressing the sympathetic nerve. The remarkable point about the case is that the disease was accompanied by *permanent contractions of the pupil.*

The circular fibres of the iris which contract the pupil so supplied by filaments of the third and fifth nerves, while the radiating fibres, which dilate it, get their nervous supply from the sympathetic filaments which unite with the fifth, in front of the Casserian ganglion. Hence stimulation of the sympathetic nerve in the neck causes dilatation of the pupil, and section of the same produces contraction, passively, by cutting off the supply of dilating power. In this case compression acts in the same manner as section. On following up this nervous connection, it becomes manifest that the sympathetic nerve does not originate the dilating power, but that it receives these motor filaments from the spinal cord between the fifth cervical and sixth dersal. This is proved by the fact that section of the spinal nerves coming from this tract, the *regio cilio-spinalis*, has the same effect as section of the sympathetic itself.

6. *Structure of the Spinal Cord and Spinal Nerves.*—In an inaugural dissertation before the University of Dorpat, by Philip Owsjannikow; the author has entered at great length on the structure of the spinal cord and spinal nerves. The conclusions drawn from the observations were as follows:—

1. That all the fibres of the spinal nerves which enter into the spinal cord are united to gangliated cells.

2. That one filament extends to each gangliated cell from the anterior spinal root, and from the posterior root; a third, a commissural one, from the other part of the spinal marrow; and in many fishes a fourth, passing from the brain, the presence of this single fibre passing to the brain, may, as the author throws out, be of moment in reference to the question of the possibility of the same fibre being both afferent and efferent in function, a position which Du Bois-Raymond thinks tenable, as judging from his experiments, though, as a rule, this power is not put into action, in his opinion. The author of the paper now considered, thinks, on the contrary, this aforesaid power is always put into use.

3. That from each cell of the spinal marrow, a filament extends to the brain, forming the white substance.

4. That the chief mass of the spinal marrow, containing fibre and cells, is a united areolar web, which being arranged in great abundance about the central canal, and furnished with numerous bloodvessels, produces the ruddy gray color of the substance which is generally supposed to be owing to pigment cells.

5. That the gelatinous substance of Rolandi is connective tissue.

6. That the cells found as well in the posterior horns as in the surrounding substance of Rolandi, are corpuscles of the united web.

7. That the cylindrical axes are of a round form, and are composed of the same substance as the nervous cells.

8. That the cylindrical axes in the gray substance are formed of a membrane peculiar to themselves, which surrounding also the nerve cells, may be separated from the fundamental mass composed of the united web.

9. That in some fishes, the cylindrical axes of the spinal cord are exposed, the cellular web in which they are placed forming no especial investment.

10. That in those fishes which have anterior and posterior spinal roots, round gangliated cells are found, sending out in various directions divided branches.—*Ibid.*

---

7. *Minute Anatomy of the Liver.*—The minute anatomy of the liver has been lately examined by Beale,* who, from his dissections and injections, comes to the following results:

1. That the essential constitution of the liver is that of a double network of minute vessels, one of capillary blood-vessels, and another of cell-containing tubes, naturally adapted to each other. Both of these sets of tubes in each lobule appear to communicate with those of the neighboring lobules in all livers excepting that of the pig; and this circumstance is connected with the fact, that in all other animals but the pig, the hepatic lobules are not isolated by intervening and limiting fibrous tissue or capsules. As to the latter position, Beale agrees with Weber.

2. That the cell-containing tubes are in all vertebrata continuous with the ultimate fine ducts of the viscus; in some cases directly so, whilst in others, as in the rabbit, and slightly in man and the dog, a fine network of the ducts themselves intervenes. The basement membrane of these tubes being, after fœtal life, incorporated with those of the capillaries, so that the secreting hepatic cells are

*Proceedings of Royal Society, June, 1855.

only separated from the stream of blood by a single intervening membrane. The cell tubules contain the hepatic cells, as also granular and coloring matter and cell débris; the cells observing no order of arrangement, as some have thought, and contrasting in size, &c., greatly with the epithelium lining the ducts, from which they are strictly separated.

3. That the fine ducts are many times narrower at the point where they are continuous with the cell tubes, than those tubes themselves; and that the larger ducts and larger interlobular ducts freely anastomose with each other.

4. That whilst the finest biliary ducts are only composed of basement membrane, that of the larger ones is more complex, containing numerous cavities; especially in the pig, which, although generally considered to be glands, are in fact reservoirs for the bile, retaining it, and bringing it into intimate relation with the abundant surrounding blood vessels, so that it may undergo requisite changes. This the author also considers to be the function of the vasa aberrantia, so named by Weber.

In this view, it will be seen that Beale considers the structure of the liver to be strikingly different from that described by Kölliker and Hanfield Jones, and assigns a different office to the secreting and epithelium cell; for, whilst the latter looks upon the cells of the ducts as chiefly forming the bile, Beale considers that they stand in relation to the hepatic cells as the columnar epithelium (lining the stomach tubes) does to the secreting cells at the bottom of them.

Beale prepared his specimens by injecting the portal vein with lukewarm water until the bile was washed out of the ducts by it, and then injecting the ducts; after which the portal vein was injected with size. The ducts were also examined in specimens hardened in alcohol, to which a solution of soda had been added, in order to render the sections transparent.

Dusch* finds that the hepatic cells are dissolved in bile and in solutions of glycocholate of soda. They are also enlarged on the addition of chloroform, according to Lereboullet, their contents becoming very clear.—*B. & F. Med. Chirurg. Rev.*, *Oct.* 1855.

* See Canstatt.

